# Establishing community mental health clinics increased the number of patients receiving care in rural Western Uganda

**DOI:** 10.3389/frhs.2023.1133770

**Published:** 2023-07-18

**Authors:** Yusufu Kuule, Andrew E. Dobson, Birungi Mutahunga, Alex G. Stewart, Ewan Wilkinson

**Affiliations:** ^1^ Church of Uganda, Bwindi Community Hospital, Kanungu, Uganda; ^2^ College of Life and Environmental Science, University of Exeter, Exeter, United Kingdom; ^3^ The Institute of Medicine, University of Chester, Chester, United Kingdom

**Keywords:** mhGAP training, community-based mental health care, rural mental health care, task sharing, mental health gap, patient retention

## Abstract

**Background:**

Mental, neurological, and substance-use disorders cause medium to long term disability in all countries. They are amenable to treatment but often treatment is only available in hospitals, as few staff feel competent to give treatment. The WHO developed the “Mental Health GAP” (mhGAP) course to train non-specialist clinical staff in basic diagnosis and treatment. At Bwindi Community Hospital, in south-west Uganda, mental health care was initially only provided at the hospital. It was extended outside the hospital in two implementation phases, initially by establishing 17 clinics in the community, run by qualified mental health staff from the hospital. In the second implementation phase staff in 12 health centers were trained using mhGAP and ran their own clinics under supervision.

**Methods:**

Using routine data the defined data variables for the individuals attending the clinics was extracted.

**Results:**

A total of 2,617 people attended a mental health care clinic in the study period between January 2016 and March 2020. Of these 1,051 people attended more than once. The number of patients attending clinics increased from 288 during the baseline to 693 in the first implementation phase then to 839 patients in the second implementation phase. After mhGAP training, about 30% of patients were seen locally by mhGAP trained healthcare personnel. The average number of mental health patients seen each month increased from 12 to 65 over the time of the study. The number of patients living >20 km from the hospital increased from 69 in the baseline to 693 in the second implementation phase. The proportion of patients seen at the hospital clinic dropped from 100% to 27%.

**Conclusions:**

Providing mental health care in the community at a distance from the hospital substantially increased the number of people accessing mental health care. Training health center-based staff in mhGAP contributed to this. Not all patients could appropriately be managed by non-specialist clinical staff, who only had the five-day training in mhGAP. Supplies of basic medicines were not always adequate, which probably contributed to patients being lost to follow-up. About 50% of patients only attend the clinic once. Further work is required to understand the reasons.

## 1. Introduction

Mental, neurological, and substance use disorders contribute about 10% (258 million) Disability Adjusted Life Years to the Global Burden of Disease in both high-income countries and low-and-middle-income countries (1). While some individuals may die from mental, neurological, and substance use disorders, and some die early from neglect of their physical health (2), the majority of those with mental illness suffer from life-inhibiting illnesses that may result in poverty and discrimination that affects future generations as well. In some countries, including Uganda, children with uncontrolled epilepsy may be prevented from attending school due to fear of other children “catching the infection” (3). Treatment of many mental illnesses is available, but may not be accessible for a number of reasons (4).

Primary health care (health care outside of hospitals) should include mental health care if it is to be accessible to those who need it (5–8). The main barriers to accessing mental health services include centralization of mental health services in hospitals, lack of available medicines, inadequate number of mental health trained staff, mental health not being a priority for policy makers, and beliefs around the causation and appropriate treatment of mental illness which result in seeking alternative treatments such as from religious and traditional healers (4, 8–11).

There is a scarcity of specialized mental health workers in many countries (5), including Uganda (12). The World Health Organization (WHO) has developed programs such as the Mental Health Gap Action Program (mhGAP), to bridge this gap by training non-specialized clinical staff in basic skills to diagnose and treat or refer those with mental illness (13). This is not about “task shifting” but “task sharing” (6), with the intention of increasing the number of staff with these basic skills. The mhGAP program has been piloted in Uganda and shown to be effective in increasing access to mental health care to some extent (14).

It has been estimated that up to 35% of Ugandans suffer from a mental disorder and 15% would benefit from treatment (12, 13). Provision of mental health care in Uganda is a component of the national minimum healthcare package, which should be available at all levels of the health system (15). It is expected to be integrated into outpatient services at hospitals and health centers to improve access to mental health care (14, 15).

Despite having a national policy framework and the encouragement of programs such as the mhGAP in Uganda, there are marked gaps in mental health service delivery in Uganda, especially at health centers (16). Mental health care is largely provided by specialist psychiatric workers at national and regional referral hospitals, with little being offered at district hospitals or health centers (17). Relatively few of the private-not-for-profit (PNFP) hospitals provide mental health care (18).

Bwindi Community Hospital (BCH) has been offering hospital-based mental health care since 2013. From January 2018 a two phased approach was adopted to expand the service into the community with monthly clinics across the catchment area. The intention was to increase access to mental health care in the area.

This study aimed to assess if the establishment of clinics away from the hospital, and training of non-specialist clinical staff from health centers in mental health care, increased the number of patients accessing mental health care and if any contributing factors could be identified.

## 2. Methods

### 2.1. Design

This was a retrospective cohort study using routinely collected data.

### 2.2. Setting

#### 2.2.1. General setting

Uganda is a land-locked East African country. In 2020 it had an estimated population of 45 million people, 84% of whom lived in rural areas and 21% living in poverty. Life expectancy at birth was 63 years in 2017 (19). Health care is provided by both government and private providers. Rural areas are mainly served by health centers supported by hospitals.

#### 2.2.2. Specific setting

The study was carried out in the catchment of BCH, which is a PNFP Hospital in Kanungu District in Southwestern Uganda. Its main catchment area covers 17 parishes, with a total population of about 70,000, living in 101 villages (20).

BCH has been providing mental health care services since 2013. Their initial focus was on providing care at the hospital, with most of this being for outpatients. From 2013 there were ongoing discussions between the BCH senior management team, the lead for mental health care and the trustees of Jamie's Fund, a small UK charity working to improve access to mental health care in Uganda (https://jamiesfund.org.uk). This resulted in the staff at BCH trying out different ways to use their resources to increase the number of clinics in the community. Once some progress had been made in two different models it was suggested we should carry out a more formal evaluation to see if the models worked.

The mental health qualified staff at BCH were 1.5 whole time equivalent psychiatric clinical officers, a psychiatric nurse and a counsellor employed by the hospital. Patients were treated as outpatients as far as possible, but when necessary disturbed patients were admitted to the acute medical ward for management. We took as our baseline, the last two-year period when all mental health care services were hospital-based.

Both oral tablets and depot injections were available to manage mental illnesses. The main diagnoses (*with the available treatments*) were: schizophrenia (*chlorpromazine, haloperidol, benzhexol*, *depot fluphenazine)* severe depression, (*amitriptyline, imipramine),* bipolar affective disorder (*sodium valproate chlorpromazine, carbamazepine)*, epilepsy (*phenytoin, phenobarbitone, sodium valproate, carbamazepine)* and alcohol use disorder (*diazepam, thiamine and vitamin B complex*). These are old, well established treatments. Compared to treatments for other diseases they are relatively cheap, but they may be too costly for people to afford.

Counselling is provided when appropriate and accessible. Educational sessions were run in the community to inform about aspects of mental illness, its treatment and ways to remain healthy.

BCH operates “eQuality”, a voluntary health insurance scheme which for a payment of about US$5 per adult per year allows the family to access subsidized health care and treatment. Other patients pay the costs out of pocket. Unlike some other health insurance schemes, patients with chronic conditions such as mental illness are allowed to be members of the scheme.

There were 12 health centers in the catchment area. Of these, four are health center IIIs (HCIII), three government run and one PNFP. At an HCIII there are supposed to be 14 staff, led by a senior clinical officer. The other eight health centers are health center lls (HCIIs), one government and seven PNFP. BCH runs two of the private HCIIs. At an HCII there are supposed to be 3 staff, led by an enrolled nurse.

MhGAP training is encouraged by the Ugandan Ministry of Health. The five-day training course was run at BCH and used the mhGAP intervention guide training materials (21). It combined didactic lectures with role plays, communication exercises and discussions. It was led by one of the experienced national trainers for mhGAP. He had also run the “training the trainers” course attended by the three other trainers who were all qualified in mental health. Twenty two nurses and midwives from health centers were trained and ten staff from BCH.

Government health centers are given a limited supply of medicine monthly, for which there is no charge to patients. HCIIIs receive a greater quantity and range of medicines than HCIIs. Patients attending the health centers run by BCH and other private providers have to pay for medicines.

In the first implementation phase, mental health clinics were established in each of the 17 parishes, and held at local health centers, churches or local administrative headquarters. The sites were selected by the clients through their 5-member parish mental health committees.

These clinics were run by mental health qualified staff from BCH once every four-weeks, travelling by motorcycle on bumpy, dusty and sometime muddy roads. The staff also visited patients at home, to provide some of the psycho-social elements of the care packages, and engaged with people in the village to raise awareness that mental, neurological, and substance use disorders could be treated. This could require two mental health staff staying for a working week in each of two sub-counties, since many of these centers were too far to travel to daily.

At each clinic, patients were assessed for their physical and psychosocial needs. Activities at the venues include talks and discussion on aspects of mental ill health and maintaining good health, and reviews of individuals. Treatments offered included counselling, medication (both oral and depot injections), discussing way to reduce stress and promote social support (including promoting social support and resettling patients who are neglected) and promoting activities of daily living and livelihood activities. Medicine refills were given if medicines were available. Mental health staff from the hospital were not permitted by hospital procedures to provide medicines at the venues in villages other than health centers, so patients were encouraged to get them from the nearest health center or pharmacy.

In the second implementation phase, after the mhGAP training, the number of clinics was reduced to 12, one in each of the health centers where non-specialist clinical staff had been trained in mhGAP. Those clinics at churches or local administrative headquarters which had been run by the staff from the hospital were closed. Additional arrangements were also made to try to ensure better availability of medicines at these health centers.

As the staff at the health centers were full time, it was expected that this would increase access to mental health care. They were also given regular mentoring by the more experienced hospital-based staff who attended each health center once a month. After a clinic, patients requiring a home visit would be seen by qualified mental health workers from BCH. The BCH team also continued to provide some of the psycho-social elements of the care packages via village visits to patients and families that required them.

### 2.3. Participants

All patients with mental, neurological and substance misuse disorders who attended BCH or outreach clinics between January 2016 and March 2020.

### 2.4. Data variables

Data was obtained from routinely collected information in health center registers and electronic databases at BCH. Data on an individual's clinic attendances was linked across time in the databases, using a combination of identifier variables, to provide patient mental health clinic histories in terms of their frequency and time intervals.

Variables collected included patients’ demographic characteristics, clinic attendance before and after establishment of mental health services in the community, year of first diagnosis with a mental illness and the type of clinic attended. Unfortunately, some data relating to home visits and other outreach work by BCH based staff in the last half of the 2nd implementation phase was lost due to an information technology problem.

Patients were counted as being a more consistent attender of the clinic in the following 12-month period if they did not have a gap in attendance of six months or more during the 12 months. They were counted as lost to follow-up if, at the end of the 12 months, they had not attended for at least the last 6 months (22).

Data taken from the main hospital databases was validated at the original point of data entry. All data from health center and mental health electronic registers were subjected to consistency checks, and discrepancies were investigated as far as possible.

### 2.5. Analysis and statistics

The data were analyzed in EpiData (version 2.2.2.186, EpiData Association, Odense, Denmark) using frequencies and proportions. Correction for the missing data in the second implementation phase was done on the assumption that the missing data were similar to those that were not missing for that period. Sensitivity analysis was done to test the robustness of the resulting estimates with regard to this assumption.

To assess the effectiveness of the service, we focused on those patients who attended the clinic more than once in one of the three time periods (January 2016 to December 2017 [baseline], January 2018 to February 2019 [first implementation phase], March 2019 to March 2020 [second implementation phase) as they had established a relationship with the clinic. Those who attended only once did not necessarily have a mental illness.

Assessing the frequency of attendance as part of this evaluation was complicated by the fact that the length of the time periods varied. For existing patients, all attendances in any of the three time periods were counted. For new patients, reattendance within six months was used to enable comparison between periods. It was assumed that the proportion of new patients who re-attended within 6 months of their first attendance was the same in both the first six months and the final six month of the second implementation phase, to compensate for missing data. Again, sensitivity analysis was done to test the estimates based on this assumption.

We investigated factors associated with patients’ repeated clinic attendance patterns in each period. For this analysis, an individual may be counted in more than one of the three time periods. Demographic and clinic characteristics of patients were compared with clinic attendance variables using multivariate logistic regression with results presented as odds ratios (OR) and 95% confidence.

## 3. Results

A total of 2,617 people attended a mental health care clinic in the study period between January 2016 and March 2020. Of these 1,051 people attended more than once. The three time periods, together with the number of clinics and who ran them, are summarized in Table 1.

**Table 1:** The time periods in the study and number of mental health clinics in the catchment area of Bwindi community hospital, Uganda.

Time period	Number of clinics	Staff running clinics
Base line, Jan 2016 to Dec 2017	1	Mental health qualified staff from hospital
Implementation phase 1, Jan 2018 to Feb 2019	17	Mental health qualified staff from hospital
Implementation phase 2, Mar 2019 to Mar 2020	12	Health centre staff trained in MhGAP and Mental health qualified staff from hospital

The characteristics of the patients who attended more than once in each of the three periods of the study are shown in Table 2. There was little difference between the study periods in the patient demographics, with about 60% being male, and about 60% aged between 18 and 59. Most were subsistence farmers.

**Table 2:** Characteristics of patients attending mental health services in the catchment area of Bwindi community hospital, Uganda between 2016 and 2020.

Characteristics	Base line, Jan 16 to Dec 17	First implementation phase, Jan 18 to Feb 19	Second implementation phase, after mhGAP training Mar 19 to Mar 20
Total number of patients (excluding first time attenders in each period who only attended once)	288	693	839
Age (in years)			
0–17	64 (22%)	143 (21%)	160 (19%)
18–35	107 (37%)	207 (30%)	267 (32%)
36–59	70 (24%)	215 (31%)	265 (32%)
60 + years	46 (16%)	125 (18%)	147 (18%)
Not recorded	1 (0%)	3 (0%)	0 (0%)
Sex			
Male	178 (62%)	450 (65%)	515 (61%)
Female	110 (38%)	240 (35%)	322 (38%)
Not recorded	0 (0%)	3 (0%)	2 (0%)
eQuality insurance status			
All of the period	112 (39%)	188 (27%)	255 (30%)
Some of the period^a^	153 (53%)	172 (25%)	175 (21%)
None of the period	23 (8%)	333 (48%)	409 (49%)
Distance from home to BCH			
Near (<10 Km)	90 (31%)	94 (14%)	88 (10%)
Far (10–20 Km)	129 (45%)	288 (42%)	298 (36%)
Very far (>20 Km)	69 (24%)	311 (45%)	453 (54%)

^a^ Patients who are only members of the “eQuality” health insurance scheme for part of a year, as they did not continue the subscription for the full year. Probably as they could not afford it.

The number of patients increased in each successive time period, from 288 to 693 to 839 patients, excluding those who attended only once. This was due to increased recruitment of new patients, as the proportion of patient retained between the periods remained the same at about 75%. The average number of patients seen each month increased steadily (Table 3). The main three diagnoses were the same in each time period, epilepsy, depression and bipolar affective disorder.

**Table 3:** Patients’ utilization of mental health services in the catchment area of Bwindi community hospital, Uganda, between 2016 and 2020.

Variable	Base line Jan 16 to Dec 17		First implementation phase Jan 18 to Feb 19		Second implementation phase Mar 19 to Mar 20	
	*N* (%)	Average monthly attendance	*N* (%)	Average monthly attendance	*N* (%)	Average monthly attendance
Total number of patients (excluding first time attenders in each period who only attended once)	288	12.0	693	49.5	839	64.5
Existing and new patients						
Patients from previous period	168 (58%)	7.0	221 (32%)	15.8	548 (65%)	42.2
New patients	120 (42%)	5.9	472 (68%)	33.7	291 (35%)	22.4
Frequency of patient attendance						
a) New patients (in 6 months from 1st attendance)						
Attended twice	50 (42%)		194 (41%)		142 (49%)	
Attended 3 times	28 (23%)	1.8	99 (21%)	12.8	70 (24%)	6.1
Attended 4 times or more	42 (35%)		179 (38%)		79 (27%)	
b) Existing patients (in full period)						
Attended once	31 (18%)		46 (21%)		137 (25%)	
Attended twice	13 (8%)		29 (13%)		88 (16%)	
Attended 3 times	10 (6%)	4.8	17 (8%)	9.2	49 (9%)	21.1
Attended 4 times or more	114 (68%)		129 (58%)		274 (50%)	
Clinical diagnosis						
Epilepsy	137 (48%)	5.7	264 (38%)	18.9	307 (37%)	23.6
Depression	59 (20%)	2.5	188 (27%)	13.4	237 (28%)	18.2
Bipolar affective disorder	25 (9%)	1.0	66 (10%	4.7	79 (9%)	6.1
Psychosis	35 (12%)	1.5	38 (5%)	2.7	56 (7%)	4.3
Child and adolescent disorder, SUD^a^ & Self-harm	7 (2%)	0.3	16 (2%)	1.1	25 (3%)	1.9
Other mental illness	23 (8%)	1.0	70 (10%)	5.0	79 (9%)	6.1
Condition not recorded	2 (1%)	0.1	51 (7%)	3.6	56 (7%)	4.3
Type of clinic attended by patient						
Hospital-based BCH-run clinic^b^	288 (100%)		229 (33%)		233 (28%)	
Community-based BCH-run clinics^b^	-		160 (23%)		38 (5%)	
Health center-based & run clinics^b^	-		-		253 (30%)	
Combinations of the above clinics	-		37 (5%)		96 (11%)	
BCH home visits only	-		267 (39%)		219 (26%)	
Distance from home to BCH						
Near (<5 Km)	90 (31%)	3.8	94 (14%)	6.7	88 (10%)	6.8
Far (5–10 Km)	129 (45%)	5.4	288 (42%)	20.6	298 (36%)	22.9
Very far (>10 Km)	69 (24%)	2.9	311 (45%)	22.2	453 (54%)	34.8

^a^ SUD, Substance Use Disorders.

^b^ Many patients were also seen on home visits.

Attendance at the different types of clinics changed markedly following the establishment of clinics outside the hospital (Table 3). Initially everyone had to attend the BCH hospital-based clinic. In the first implementation phase, when services were provided by staff from BCH working in the community, only 229/693 (33%) were seen at the hospital and the rest seen either in the village 267/693 (39%) or at clinics 160/693 (23%). A few (5%) were seen in more than one place.

In the second implementation phase, after the mhGAP training, only 233/839 (27%) of patients were seen at the hospital, and 253/839 (30%) were seen by the mhGAP-trained health center staff. The remainder were seen by BCH staff in the village 218/839 (26%) or a community clinic 38/839 (4%) with 96/839 (11%) seen at more than one location.

During both the first and second implementation phases, many of the patients seen at a community-based clinic run by staff from BCH or a lower Health Center were also seen during a home (or village) visit, reflecting the fact that the BCH team was continuing to provide some of the psycho-social elements of the care.

The number of patients attending clinics who lived more than 20 km from BCH was six times higher in the second implementation phase (following the mhGAP training) than in the baseline period. The number who lived <10 km from BCH was similar changed very little. At the same time the proportion of patients not enrolled in BCH's eQuality health insurance increased from 8% to 48% and 49% in the first and second implementation phases.

In the second implementation phase, following the mhGAP training, three of the twelve health centers saw the majority of the patients (69%) seen at Health Centers. Two were over 20 km from BCH and the other one was just under the 20 km.

Around half of the patients attending the service only attended once: 46% (241) before clinics were established outside the hospital, 54% (829) in the first implementation phase, and 37% (496) in the second implementation phase, following the mhGAP training. Many of these were diagnosed with a mental illness but we have no data as to why they did not re-attend.

Regular clinic attendance is important to maintain a supply of medicine and support. Around 30% of new patients attended four or more times in the first six months, while over 50% of those who had continued to attend from the previous period attended four or more times in the next period (Table 3).

Factors associated with a patient being a more consistent attender of the clinic in the following 12-month period (i.e., neither lost to follow up nor having a gap in attendance of six months or more during the period) were: being aged between 18 and 59, having eQuality insurance (both *p* < 0.001), having psychosis or epilepsy rather than one of the other mainly shorter-term conditions (*p* < 0.01).

## 4. Discussion

This study shows that in a poor, very rural area of a low income country, staff with very limited resources succeeded in substantially increasing the number of individuals attending mental health clinics. This was sustained over a two year period by opening clinics in the community and training health centre staff in mhGAP. Furthermore, this was done as part of normal service development, with only the costs of the two mhGAP courses being sponsored externally, but with supportive senior management and well motivated staff. We have not found any other studies describing this achievement.

About half of all the patients attending the service in each of the three study periods only attended once, and a further significant proportion were lost to follow up. A Rwandan study found 35% of patients only attended once (23). This is about two thirds the rate in our study.

Elsewhere in Uganda (14) an increase in patient numbers was seen in a six month pilot study when health center staff were trained in mhGAP. That study was generously funded by an external donor. They report an increase of 35% (670 to 1016) in attendance in the six months follow the training. However, their data is based on that reported the Ugandan National Health Information Management System. This only records attendances, not cases, so although the numbers increased it is not clear if any patient attended more than once. The increase they describe would be accounted for by 346 patients attending one extra time in the second six months. Due to their data source, there is no information on patient retention or frequency of attendance.

Until we analyzed our data for this study, we were unaware of the high number of patients attending only once, as previously the data focus was counting the number of patient attendances, as required by the Ugandan National Health Information Management System.

This shows the importance of both collecting the relevant data and also analyzing it. The data to show that many patients who would benefit from treatment were not attending regularly, or even more than once, had been collected by the mental health services at BCH, but not used. As has been said elsewhere “We are drowning in information but starved for knowledge” (24).

Around 30% of new patients attended four or more times in the first six months. This is likely to be adequate for them to have enough medication to be able to take it regularly, but we have no data on concordance with medication.

One of the challenges accessing health care when living in a rural area is the distance to the nearest appropriate facility (25). The main increase in patient numbers accessing mental health care came in those living at a distance from BCH. As stated above, the number living more than 20 km from the hospital increased six-fold over the study period, while the percentage of patients from more than 20 km doubled: from 69 (24%) to 453 (54%). This is probably simply because the service became easier to access for those living further from the hospital. This finding is similar to the study in Rwanda (23) which reported that the majority of patients attended health centers which were <5 km from home, rather than travelling further to the main clinic at the hospital.

In many low income countries, access to a reliable, affordable supply of appropriate medicines is a major barrier to mental health care (9, 11, 26). This was certainly a problem in our study. Government HClll are supposed to receive a monthly stock of psychotropic medication so may have slightly more reliable supplies of free medication. The private HClls have to charge for medication and often do not have adequate supplies. The two HClls run by BCH offered subsidized mental health medication through the eQuality health insurance scheme but this requires families to have enough money to be part of the insurance scheme (27). It is discouraging for patients to make the effort to attend a clinic to be told their medication is out of stock, or it may be that they cannot afford the ongoing expense of the medication when attending private health centers. This is likely to contribute to the loss to follow up.

Three of the twelve health centers saw the majority (239, 69%) of the patients reviewed by health center staff in the second implementation phase. These were two government HCllls and one private HCll run by BCH. The reasons for this higher demand are not known. Contributing factors may be the availability of medicines, which were free at two of the clinics, the greater number of trained staff at a HCIII, or the level of enthusiasm and/or confidence of the staff. Population density or accessibility were not appreciably different from other clinics. Anecdotally, some of the health center staff did not consider it part of their role to provide care for those with mental illness and have been known to request payment per patient from their employer for them to do this. This was also found elsewhere in Uganda (14).

People were more likely to continue to attend a clinic if they had a major mental illness or epilepsy and were of working age, or were enrolled in BCH's eQuality insurance, suggesting that cash-flow or access to funds is an important determinant of access to continuing care. For those with a major mental illness or of working age, the beneficial effect of medication probably gave an adequate incentive to continue to take the medicine. Those with epilepsy may be more likely to continue to attend the clinic as the positive impact of treatment is more marked and may be more valued. Others have found that some people with mental, neurological or substance use disorders, live socio-economically and psychologically chaotic lives, so attending a clinic may not be a priority (28). Those with depression in India were found not to see depression and anxiety as necessarily being suitable for medical treatment (29). This has been described for all mental illnesses in Uganda (30). An interesting paper from Ghana (31) describes how many people with psychosis discontinued their medication as they found the side effects such as drowsiness, restlessness, and movement abnormalities unpleasant and these conflicted with concepts of health, healing, and wellness. The patients felt that failure to achieve a permanent cure cast doubt on the medication's long term efficacy and further suggested to them that spiritual factors might also be at play. This may well also true in Uganda.

The percentage of people with eQuality insurance fell over the three periods, although the total number insured increased. This was not surprising as it has been observed previously (32) that people living closer to BCH are more likely to be in the insurance scheme. As more people living further from the hospital attended the new clinics they would be less likely to be part of the insurance scheme. However, access to effective treatment may have contributed to the increased number of patients joining the insurance scheme. Encouraging mental health patients to enroll in the scheme could possibly improve compliance with treatment and be worth pursuing.

The number of new patients fell in the second implementation phase while the number retained increased. This might mean that there were fewer people left who needed to attend the clinic.

The Programme for Improving Mental Health Care (PRIME) was an international programme researching ways to improve mental health care (33). They have worked in five countries and recommend a comprehensive planning approach. However, even this does not guarantee success, as they reported that in Uganda, India and Ethiopia they found a small non-significant improvement in treatment coverage of depression and a significant improvement in diagnosis of depression and alcohol use disorder, but this improvement was not sustained over the longer term in Uganda (34).

Their Ugandan study was undertaken on a much larger scale than ours, with multiple focus groups and coordination groups, and yet the impact was not sustained. We found better long term results in increasing treatment coverage in a program run without extra (external) funding. Introducing service developments with motivated local leadership, using existing funding, and which can be evaluated from routinely collected local data, must be a more appropriate and sustainable approach than the studies that are often published. Such published studies often depend on significant, but short term, international funding to “pilot” a model, which may have been shown to work elsewhere already. The conclusion of these “pilots” is too often that the model should be implemented on a wider basis in that country. But that expansion usually has to await further external funding, which often is not forthcoming. PNFP hospitals may be able change service models more easily than government hospitals, as they usually have more autonomy, flexibility and, perhaps, more motivated staff.

A study in Malawi (35) where 43 primary health care workers were trained for two days in mhGAP found that there was an increase in the numbers of patients with a diagnosis of mental illness attending the 18 health centers but the numbers were very low, increasing from a total of about 15 cases per month pre-training to a total of about 31 per month. It is not clear if these are cases or patient-contacts. This is a much smaller number and increase than we found (Table 3).

One of the benefits of this provision of mental health care in the community was seen during the subsequent COVID-19 lockdowns, as patients were able to receive care from these health centers even when staff from BCH were limited in their travel.

Strengths of this study include that it has been carried out using routine data, much of which has been collected electronically, which is unusual in a very rural poor community in much of Africa. It is unlikely that we could have undertaken this study with paper-based records. Also, the clear step-changes in attendances in the two implementation phases and the fact that data was collected over several years give credence to the findings.

Weaknesses include the following. We were only able to report on attendances by individuals but do not know the accuracy of the diagnoses or the reasons for people not returning to the clinic. Some of the data on clinical workload in the community was lost due to an IT issue. Both these weaknesses reflect that this project was operational research carried out in rural Africa in a small non-academic hospital without external funding.

Analysis of what treatment was prescribed has not been done due to lack of resources, nor do we have data on when and how much medication patients received. This would have enabled us to see if they had enough medication to take at the recommended dosage until their next appointment. Outcome data, particularly in terms of control of their illness would have been helpful to assess the effect on peoples’ lives. In the other studies as part of the PRIME initiative (34) they recruited full time staff to assess the impact of treatment. We did not have the resources to do this.

### 4.1. Practical implications

Providing more clinics in the community, nearer where people live resulted in many more patients attending mental health care clinic which was continued over time.

Training non-specialist staff in mhGAP adds to the workforce able to diagnose and treat or refer people with mental health conditions (36). This increases the likelihood that patients will access care. Care for mental ill-health is much more accessible if it is provided by staff based in a health center each day, not just at a monthly clinic. This has been achieved at the minimal cost of running two mhGAP training courses with the motivation and supervision coming from the mental health qualified staff in the hospital as part of their service development work.

These non-specialist staff complement, rather than replace, the mental health qualified staff, who are still required. It is not appropriate that all the care of patients with a mental illness should be transferred to non-specialist clinicians who have undergone a five-day training in mhGAP. There is still a need for mental health-qualified staff to review and manage some of the more complex patients, though the workload of the mental health staff has been reduced as they no longer have to see all patients. Due to data loss, this reduction in workload cannot be reported in detail.

BCH has relatively well-developed electronic records, with data from both its hospital and community programs facilitating this retrospective study. We recommend that the hospital systems are refined to enable routine data to be used to identify patients’ failures to attend a clinic on a real-time basis. This could significantly improve the standard of service provided to those with mental illness and other non-communicable diseases that benefit from regular follow up. Text messages could then be used to remind patients before their appointments, or to check with patients if they do not attend. This is already used in other patient programs.

Gathering data routinely on the quantities of drugs prescribed and dispensed would enable a much clearer understanding of how patients were using the service. We do not have data on what medication patients actually received as opposed to what was prescribed, so we have little idea if they were able to take their medicines continuously. As the routine treatment of all non-communicable diseases becomes more established, it would be helpful to be able to monitor more effectively how well patients are being managed in routine care.

Further qualitative research would be helpful to understand why the loss to follow up rate was so high. It could be due to socioeconomic factors and the cost of medication from some clinics, local health beliefs and expectations, (such as an expectation of a cure rather than long term medication) and/or the stigma that remains around mental health disorders.

### 4.2. Lessons learned

Robust backup systems for electronic data and appropriate training are important. We thought there was a good system in place to back up our data but we still managed to lose six months of data that had been entered on the system which recorded the work the hospital staff had done in the community.

## 5. Conclusion

We have shown that providing community mental health care in a rural Ugandan setting resulted in substantially higher numbers of patients both starting and continuing to attend clinics. We also identified that a large number of our patients only attend a mental health clinic once or twice and do not return. This requires further research to understand the reasons for this loss and to help develop appropriate services to enable this disadvantaged group to receive appropriate treatment.

## Data Availability

The raw data supporting the conclusions of this article will be made available by the authors, without undue reservation.
